# Quantifying ventilatory control with 3% CO2 inhalation during exercise

**DOI:** 10.3389/fphys.2025.1528519

**Published:** 2025-04-25

**Authors:** Suhaib M. Hashem, Stanley M. Yamashiro, Takahide Kato, Takaaki Matsumoto, Vasilis Z. Marmarelis

**Affiliations:** ^1^ A. E. Mann Department of Biomedical Engineering, University of Southern California, Los Angeles, CA, United States; ^2^ National Institute of Technology, Toyota College, Toyota, Japan; ^3^ School of Health and Sport Sciences, Chukyo University, Toyota, Japan

**Keywords:** exercise ventilatory response, hypercapnic ventilatory response, dynamic modeling and analysis, LTI system identification, end tidal capnometry (EtCO2)

## Abstract

**Introduction:**

CO2 mediated ventilation is mainly controlled by two homeostatic mechanisms. The central chemoreceptors are slower mechanisms that focus on blood pH sensing in the brain stem while the peripheral chemoreceptors are quicker to respond and reside in the carotid bodies. Quantification of these mechanisms in humans remain debated.

**Objective:**

To quantify the impact that the central and peripheral chemoreceptors have on ventilation in response to changes in PETCO2 during exercise with normoxic breathing and 3% CO2 inhalation.

**Method:**

Six healthy males participated in a 5-stage bike protocol with and without 3% CO2 inhalation. We analyzed the time series data of their breath-by-breath PETCO2 and ventilation and generated a one input–one output model via the Laguerre expansion technique (LET) to construct the gain function and quantify the low (0.002–0.029 Hz) and high (0.03–0.15 Hz) frequency components using the weighted gain averages (WGA) as estimators of central and peripheral chemoreflex mechanisms respectively.

**Results:**

3% CO2 inhalation caused a significant increase the high frequency WGAs at rest and in all levels of exercise except heavy exercise. The low frequency WGAs, however, only maintain significance during rest and the baseline session of exercise.

**Conclusion:**

Changes in WGA can be used as quantitative estimates of central and peripheral chemoreflexes. 3% CO2 activates both reflexes and is more apparent in the higher frequency WGAs during exercise due to the oxygen dependent mechanisms effects of exercise.

## Introduction

It has long been accepted that two major receptors exist in the control of ventilation to regulate PaCO2 and maintain relatively constant levels of oxygenation while disposing of CO2 through ventilatory action: the peripheral (carotid) and central (medullary) chemoreceptors (Dahan et al., 2007; Dahan et al., 1990; Heymans, 1930; Nattie and Li, 2012). The central chemoreceptors typically respond slowly to changes in CO2 (i.e., blood pH) whereas the peripheral chemoreceptors respond much more quickly to O2 and CO2 (Pittman, 2011; Yang and Khoo, 1994; Pedersen et al., 1999). Exercise modulates the effect of these chemoreceptors to match the metabolic demand of the body (Clark et al., 1980; Dempsey et al., 1984; Casey et al., 1987). Studies have shown that in carotid (peripheral chemoreceptor) resection, subjects lose their ability to control ventilation due to hypoxia and have a reduced ventilatory response to hypercapnia by one-third (Wasserman et al., 1975). There is a debate as to which chemoreceptors are more responsible in mild exercise in comparison to heavy exercise. This mainly stems from the differing analytical methods used to quantify the strength of the chemoreflexes (response of the chemoreceptor systems).

Many experiments took to different methods to measure the ventilatory response to induced CO2 during exercise. Varying results have been observed due to the variable levels of CO2 administration (Berkenbosch et al., 1989; Savulich et al., 2019; Duffin et al., 1980; Read and Leigh, 1967). Some experiments that have relied on rebreathing showed no difference in CO2 sensitivity in exercise (Duffin et al., 1980). Constant high levels of CO2 inhalation (7.5% or more) have been shown to increase arousal and anxiety (Savulich et al., 2019). Lower levels of CO2 inhalation have generally been avoided due to the lack of a response that can be measured from the subjects. Recently, it has been previously shown that peripheral and central chemoreceptor augmentation can be sufficiently observed with 3% CO2 inhalation during mild exercise (Yamashiro et al., 2021). However, it has been difficult to model the dynamics of short single sessions to compare how ventilatory regulation changes in response to different levels of exercise.

Dynamic modeling methods have long been utilized to understand the relationships between different physiological systems including, but not limited to, the cardiovascular and respiratory systems (Yamashiro et al., 2021; Marmarelis et al., 2017; Mitsis et al., 2009; Grodins et al., 1954; Khoo and Marmarelis, 1989). From among these methods, we have opted to use the Laguerre Expansion Technique (LET) to take advantage of its ability to model relatively short and noisy data with high accuracy, as well as its independence from parameter assumptions (Marmarelis, 1993; Marmarelis, 2004). This study aims to quantify the impact of central and peripheral chemoreceptors to ventilation changes during mild and heavy exercise in response to PETCO2 variations under normoxic and 3% CO2 inhalation conditions.

## Materials and methods

### Experimental protocol

Six healthy male subjects (age 21.8 ± 0.4 years; height 170.88 ± 7.2 cm; body mass 65.8 ± 3.8 kg; VO2max 43.1 ± 6.1 mL/kg/min; mean ± SD) with no history of cardiorespiratory diseases participated in a five-stage cycling protocol twice, once while breathing normoxic air and once with 3% CO2 inhalation. The protocol commenced with a 6-min baseline resting period (session 1), followed by 6 minutes of baseline exercise at 40 W (session 2). The intensity was then increased to 40% of their maximum oxygen uptake (VO2 max) for another 6 minutes (session 3). This was followed by a return to 6 minutes of exercise at the baseline intensity (session 4). The final stage consisted of 6 minutes of intense exercise at 80% of VO2 max (session 5). Each subject was consented in compliance with the Human Subjects Committee at the Chukyo University Graduate School of Health Sciences. Data was collected on a breath-by-breath basis of all relevant ventilatory variables. This includes but is not limited to, end-tidal CO2 (PETCO2), tidal volume, and respiration rate. The specifics of the exercise protocol and the VO2 max measurement procedures are detailed in the Kato and Yamashiro study (Yamashiro et al., 2021; Yamashiro et al., 2024).

### Data preprocessing

Each subject’s breath-to-breath data was preprocessed separately for each session by removing the DC value and very slow trends (<0.005 Hz) through high-pass filtering from evenly sampled data at 1 Hz (via interpolation). The preprocessed data was then clipped at ±3 standard deviations.

### Modeling methodology

The preprocessed data was used to analyze the dynamic relationship between the PETCO2 (input) and ventilation (output) by estimating the Impulse Response Function (IRF)/kernel via the Laguerre expansion technique (LET) (Yamashiro et al., 2024; Murias et al., 2014). A linear one-input one-output model can be described using the generalized Volterra series:

$$y\left(t\right)=k_{0}+\int_{0}^{\infty }k_{1}\left(\tau \right)x\left(t-\tau \right)d\tau$$

Here, y(t) and x(t) represent the PETCO2 and ventilation time series data, respectively. The kernel (k1) denotes a canonical representation of the system dynamics for a given input-output relation. To minimize the estimated parameters and allow for the use of relatively short noisy data, we expand the kernel into a series of coefficients and a set of orthogonal basis functions (discretized Laguerre polynomials). By convolving the input data with the basis functions, we can solve this linear relationship by utilizing the Least Squares Method:

$${\left(X^{T}X\right)}^{-1}X^{T}y=c_{j}$$

In this equation, X is the input data convolved with the Laguerre basis functions and y is the output data. The variable $c_{j}$ is the array of coefficients of the *j*th Laguerre function. The number of Laguerre functions is selected by using the Bayesian Information Criterion. For this model, we used four Laguerre functions. The calculated kernel in the provided equation provides a quantifiable method to understand how the output (minute ventilation) is modulated by changes in the input (PETCO2).

The model is evaluated for its accuracy by calculating the Normalized Mean Square Error (NMSE) using the following equation:

$$\left.NMSE=\right\|||y_{pred}\left.\left(t\right)-y\left(t\right)\right|{|}^{2}\left./\right|\left|y\left(t\right)\right|{|}^{2}*\;100$$

where y_pred_ is the predicted output generated from the calculated coefficients. The double bars (||.||) denote the sum of squares for the values between them.

### Weighted gain averages (WGA)

To enhance our understanding of the Impulse Response Function (IRF) dynamics, we computed the Gain function, which represents the magnitude of the IRF spectrum. The Gain function was calculated across all sessions, and the weighted average was determined using the CO_2_ input power spectrum. The central and peripheral chemoreflex mechanisms were assessed by identifying frequency ranges from 0.002 to 0.029 Hz (central chemoreflex) and from 0.03 to 0.15 Hz (peripheral chemoreflex), respectively. We calculated the weighted average of the Gain function for each spectral range, factoring in the CO2 input power spectrum, using the following formula:

$$\text{WGA}=\text{Average}\left\{G\left(f\right)\;X\left(f\right)\right\}/\text{ Average}\left\{X\left(f\right)\right\}$$

Here, G(f) and X(f) represent the Gain function and the PETCO2 input spectrum, respectively, at each frequency range *f*. Averages were computed across the previously specified low and high frequency ranges. These newly calculated indices allow us to quantify the impact on ventilation that PETCO2 has within each frequency range; normalizing by the PETCO2 spectrum allows for a comparative physiological marker between subjects.

### Statistical analysis

We began by performing a two-way repeated measures ANOVA to assess whether exercise intensity influenced the effect of inhaled CO2. The significance of the WGAs were then evaluated for each subject by conducting a paired t-test to observe the difference between normal air and 3% CO2 inhalation sessions.

## Results

The means and standard errors of the time-average (DC) PETCO2 and ventilation values, as well as the averages of the NMSE for each session, are presented in Table 1 with their respective paired t-test p-values. All PETCO2 and ventilation difference values maintain significance throughout each session (p < 10^–4^ and p < 10^–3^, respectively). A two-way repeated measures ANOVA also revealed that gas inhalation and exercise intensity were both significant main effect predictors for both PETCO2 (p < 0.001) and ventilation (p < 0.01) with no interactions between the two conditions. NMSE on the other hand did not have a main effect predictor but instead exhibited an interaction between both conditions (p < 0.001).

**TABLE 1:** Mean (SE) of end-tidal CO2, ventilation, and NMSE of each session for both conditions (normoxic breathing and 3% CO2 inhalation) with p-values of paired t-tests across conditions (significant p-values in bold).

Session number	End-tidal CO2 (mmHg)		Ventilation (L/min)		NMSE (%)	
	Normal air means (SE)	3% CO2 means (SE)	Normal air means (SE)	3% CO2 means (SE)	Normal air means (SE)	3% CO2 means (SE)
1	36.35 (1.23)	42.45 (1.72)	12.15 (0.83)	14.96 (1.29)	67.80 (7.11)	34.01 (6.42)
p-value	1.33*10 ^**−** **4**^		**0.0023**		**0.0267**	
2	42.05 (0.45)	48.02 (0.47)	24.24 (1.24)	31.25 (1.85)	44.73 (3.83)	41.43 (3.54)
p-value	3.98*10 ^**−** **6**^		**0.0033**		0.200	
3	43.69 (0.91)	51.03 (0.82)	33.87 (0.79)	43.86 (1.37)	41.61 (7.94)	47.22 (5.27)
p-value	6.42*10 ^**−** **6**^		2.49*10 ^**−** **4**^		0.3344	
4	40.35 (0.50)	47.14 (0.53)	30.28 (1.04)	40.10 (0.92)	57.02 (2.52)	57.06 (3.28
p-value	4.69*10 ^**−** **5**^		1.67*10 ^**−** **5**^		0.9894	
5	42.18 (1.16)	52.45 (0.90)	66.19 (1.85)	79.72 (2.27)	41.43 (6.20)	63.02 (4.96)
p-value	9.40*10 ^**−** **5**^		**0.0030**		**0.0085**	

**FIGURE 1:**
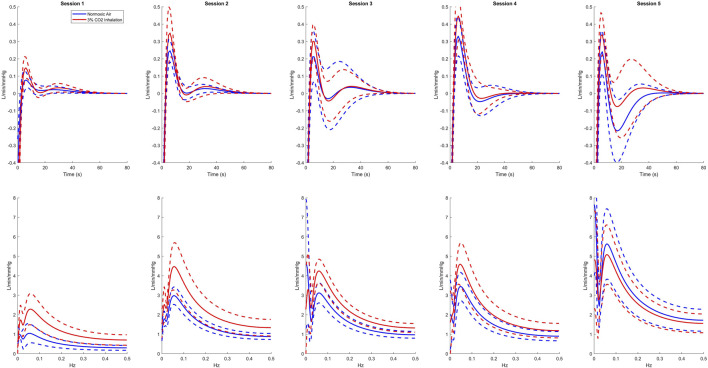
Average kernel estimates (top) and gain functions (bottom) of all sessions for normoxic breathing (blue) and 3% CO2 inhalation (red). Dashed lines represent one standard deviation above and below the mean.

**FIGURE 2:**
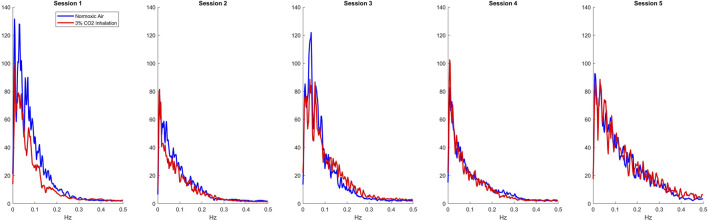
Average input Spectrums of all sessions for normoxic breathing (blue) and 3% CO2 inhalation (red).

Using the LET, we created plots of the IRF’s for each session as presented in the top row of Figure 1. For all sessions, 3% CO2 inhalation increases at around the 5 s lag mark. There are also small negative values shown around the 20 s lag mark followed by a slight overshoot around the 30 s lag mark. This, however, is difficult to separate between the two groups and hence leads us to observing the Gain function and calculating the WGA described above. Analysis of the Gain function revealed two resonant spectral peaks, postulated to be the central and peripheral chemoreflex mechanisms, as seen on the second row of Figure 1. The central chemoreflex was observed within the frequency range of 0.002–0.029 Hz, where a notable trough occurred, and the peripheral chemoreflex was observed between 0.03 and 0.15 Hz, as seen in Figure 1. The PETCO_2_ input signal showed minimal power above 0.15 Hz (Figure 2). The WGAs were calculated by averaging and normalizing for each respective spectral range.

In a two-way repeated measures ANOVA, the analysis revealed that for the low-frequency WGAs, exercise was a significant main effect predictor (p = 0.0168), whereas gas level was not (p = 0.3423). Conversely, for the high-frequency WGAs, both exercise and gas level were significant main effect predictors (p < 0.01), but they did not interact (p = 0.3338). Table 2 details the resulting WGA for both frequency ranges as well as their respective differences and paired t-test p-values. Examining the WGA for the various sessions/conditions, we observe that 3% CO2 inhalation in session 1 causes a significant change in the slower (central chemoreflex) and faster (peripheral chemoreflex) dynamics. The difference in the slower dynamics loses significance as soon as the 45% VO_2_ max exercise is introduced. This can be possibly due to a saturation of the amount of CO2 that drives the central chemoreflex. It is also evident that for the slower dynamics, once the subjects return to baseline exercise, the WGA for the normal air breathing group drops but not to a significant degree (p = 0.2527). This may be due to the time it takes for the central mechanisms to relax or desaturate from the increased CO_2_ introduced in the earlier session. The faster dynamics maintain a significant difference through all sessions, with the exception ofsession 5 (p = 0.8503).

**TABLE 2:** Weighted Gain Averages (WGA) of each session for both conditions (normoxic breathing and 3% CO2 inhalation) with p-values of paired t-tests across conditions (significant p-values in bold).

Session number	Low frequency WGA (L/min/mmHg)		High frequency WGA (L/min/mmHg)	
	Normal air means (SE)	3% CO2 means (SE)	Normal air means (SE)	3% CO2 means (SE)
1	0.86 (0.11)	1.53 (0.24)	0.86 (0.18)	2.01 (0.29)
p-value	**0.0364**		**0.0170**	
2	1.65 (0.08)	2.38 (0.24)	2.51 (0.16)	3.82 (0.43)
p-value	**0.0386**		**0.0253**	
3	2.69 (0.73)	2.80 (0.50)	2.54 (0.11)	3.59 (0.20)
p-value	0.8476		**0.0021**	
4	1.80 (0.30)	2.36 (0.26)	2.78 (0.25)	3.81 (0.39)
p-value	0.2527		**0.0039**	
5	4.23 (0.60)	3.57 (0.75)	4.25 (0.43)	4.31 (0.50)
p-value	0.4746		0.8503	

**FIGURE 3:**
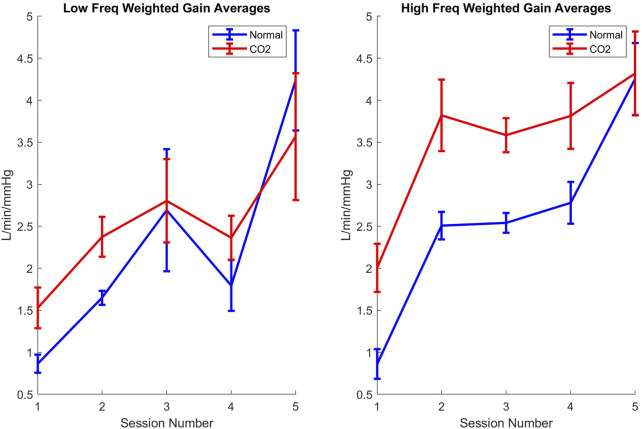
Line plots of weighted gain averages throughout the sessions for low (left) and high (right) frequency mechanisms for normoxic (blue) and 3% CO2 inhalation (red). Error bars represent standard error.

As depicted in the line plots in Figure 3, exercise has a less significant impact on the trends of WGA at lower frequencies compared to its more pronounced effects at higher frequencies. Furthermore, at these higher frequencies, the influence of CO_2_ and heavy exercise on WGA closely resembles the outcomes observed during the anaerobic exercise level (Session 5) in the normal air breathing protocol.

## Discussion

This study investigated the dynamic responses of ventilation to PETCO2 under the influences of 3% CO2 inhalation during varying levels of exercise intensities using the LET. This study utilizes the WGA to separate these effects in each session so that each level of exercise can be studied individually. Traditional modeling methods, due to the brief durations of the experimental sessions, would require a longer data record than available in a single session. The LET, in contrast, allows for a detailed understanding of dynamics even with shorter record lengths, making it particularly suitable for our study design.

The notable increase in the WGA for both central and peripheral chemoreflexes makes clear the substantial impact that CO2 inhalation has on these mechanisms. The central chemoreflex is widely known as the main CO2 sensor in the body that controls ventilation (Heymans, 1930). This is evident by the increase in the WGA in session 1 and session 2. During exercise, the metabolic effort increases CO2 production which contributes to an increase in ventilation (Murias et al., 2014). There is, however, a limit to how much the central chemoreflex contributes to an change in ventilation from inhaled CO2 during exercise. The lack of significance in session 3 could be attributed to a saturation of CO2 in the central mechanisms due to exercise, which would no longer produce a noticeable increase in ventilation despite additional CO2 inhalation (Putnam, 2010). Kuwaki has shown that central chemoreceptor response is state dependent and that lower levels of PETCO2 induce a larger change in ventilation than higher levels (Kuwaki et al., 2010). This saturation highlights the nonlinearities that exist within the chemoreceptor response (Mateika and Duffin, 1994). In session 4, however, we notice a marked decrease in the WGA at low frequencies for both conditions, pointing to a desaturation of CO2 from the central chemoreceptors. Therefore, we conclude that heavy exercise (session 5) leads to an increase in WGA for both conditions (Jeyaranjan et al., 1987).

An increase in peripheral chemoreflex has been previously reported with CO2 inhalation during exercise (Yamashiro et al., 2021). In this study, we have been able to show that for CO2 inhalation during rest and mild exercise, there is a notable increase in peripheral chemoreflex activation than those who breathe normal air during these sessions. The peripheral chemoreceptors at the carotid bodies have been shown to be mainly sensitive to acute O2 changes. This effect is bolstered with the presence of hypercapnia (Kuwaki et al., 2010). In all sessions of light exercise, peripheral chemoreceptor response is maintained at the same level, indicating that all the tested ranges of aerobic exercise stimulate the carotid bodies in a similar way. In the heavy exercise session (Session 5), the WGA for both breathing conditions increases but they lose the significance of their difference. This loss in significance is due to exercise being the only main effect predictor as reported in the two-way repeated measures ANOVA. Session 5, when exercise is pushed to 80% or more of the VO2 max, is widely considered to be anaerobic level exercise (Davis et al., 1976). Anaerobic exercise changes the mechanism of CO2 production in the body to a non-oxygen dependent pathway (Bangsbo et al., 1990). This likely indicates that the saturation that is induced in anaerobic exercise does not allow an increase of peripheral chemoreflex sensitivity of inhaled CO2 over normal air due to the body not utilizing oxygen for exercise at this stage (Duffin and McAvoy, 1988).

The proposed methodology in this study utilizes the LET to model the complex dynamic relationships of CO2-driven ventilation. The use of this nonparametric method allows for the understanding of physiological systems without the need for predefined assumptions. Traditional modeling methods (Cunningham et al., 1986; Bellville et al., 1979; Duffin, 1972; Hey et al., 1966; St Croix et al., 1996; Duffin et al., 2000), while seminal in their work and contributions, suffer from a dependency of measurement conditions with *a priori* parameters that assume homogeneity in the subject population. The LET can model sessions of spontaneous breathing as well as sessions of different stimulations the same way. It also does not make any assumptions of the physiological systems studied. This allows for a robust and canonical representation of each subject that is solely dependent on their measured data. While this method clarifies our understanding of these respiratory regulation systems, it is important to remain vigilant of each model’s NMSE. The NMSE reveals how much of the output variance is not explained with the proposed model (Marmarelis, 2004). Table 1 showcases that in Session 1, when the subjects were inhaling 3% CO2, we have a significantly higher model accuracy (p = **0.0267**), indicating a greater contribution from the kernel (and the Gain function by extension) to observed changes in ventilation during the 3% inhaled CO2 trials. This relationship, however, is diminished when anaerobic exercise is introduced indicating that PETCO2 contributes less to the change in ventilation during heavy exercise. It is possible that the lactate threshold plays a role here, as anaerobic conditions shift the metabolic pathways, reducing the sensitivity of both central and peripheral chemoreflexes to CO_2_ (Andrade, 2025). In particular, the rise in lactate and accompanying metabolic acidosis during high-intensity exercise may shift the ventilatory control mechanisms away from CO_2_ regulation, potentially contributing to the diminished impact of inhaled CO_2_ on ventilation during these conditions. This could explain the observed increase in NMSE in Session 5, as the model’s ability to account for changes in ventilation becomes less effective under anaerobic conditions. With the shift toward anaerobic metabolism and the dominance of lactate-induced ventilation changes, the contributions from CO_2_ to ventilation become less predictable, thus leading to a larger unexplained variance in the model and an increase in NMSE for that session.

By examining the weighted gain average (WGA), we were able to identify the contribution of the central peripheral chemoreflex under different conditions. We found that the central chemoreflex response to inhaled CO2 is significantly greater than regular air at rest and during baseline 40 W exercise (p < 0.04). However, this response is reduced during mild exercise (45% VO2 max), possibly due to CO2 saturation at the central nervous system (CNS). In contrast, the peripheral chemoreflex maintained its sensitivity to inhaled CO2 at all levels of exercise (p < 0.05) until anaerobic conditions were reached, at which point the difference in response was diminished. While exercise significantly contributes to the activation of both central and peripheral chemoreflexes, inhaling CO_2_ during exercise primarily affects the activation of the peripheral chemoreflex.

## Data Availability

The datasets generated and analyzed during the current study are available from the corresponding author upon reasonable request.
